# Ultrasound-Guided Posterior Tibial Nerve Block for Frostbite of the Plantar Surfaces: A Case Series

**DOI:** 10.5811/cpcem.2022.7.56727

**Published:** 2022-10-24

**Authors:** Taylor Burl, Parker Latshaw, Andrea Dreyfuss

**Affiliations:** * Hennepin County Medical Center, Department of Emergency Medicine, Minneapolis, Minnesota; † University of Minnesota Medical School, Minneapolis, Minnesota

**Keywords:** frostbite, ultrasound-guided nerve block, posterior tibial nerve block, case series

## Abstract

**Introduction:**

Frostbite is a painful condition that requires rapid identification and wound care to optimize outcomes. The posterior tibial nerve (PTN) block, however, has yet to be described in the literature for pain control of frostbite injuries on the plantar surfaces.

**Case Series:**

In this case series we discuss three patients who presented with bilateral frostbite on the plantar surfaces. Ultrasound-guided PTN blocks were performed on these patients and pain control was achieved in under 10 minutes, facilitating burn care. No patient experienced adverse effects. All patients had been scheduled for future debridement that was either not performed or performed using intravenous (IV) medications due to pain control issues.

**Conclusion:**

The ultrasound-guided PTN block facilitated proper wound debridement that was previously intolerable with oral and IV pain medications. This case series highlights the efficacy, safety, and accessibility of this block for frostbite pain control in the emergency department. Additionally, it emphasizes the potential role of ultrasound-guided PTN blocks as part of a multi-modal pain control strategy in other clinical settings.

## INTRODUCTION

Frostbite injuries exist on a spectrum but have one thing in common: they are painful and debilitating. Peripheral nerve blocks for frostbite have been described mostly in the context of military medicine, where simple and effective pain control is needed in a prehospital setting.^1^,^2^ The PTN block is effective for pain control in distal foot amputations, surgeries, foot fractures, and foreign body removal.^3^–^8^ In the civilian setting, the emergency department (ED) is often the first point of care for patients with frostbite, where there is a need for safe, effective, and timely management of frostbite injuries to prevent long-term consequences such as chronic pain, necrosis, and amputation.^9^ This case series presents ED patients with frostbite on bilateral plantar surfaces who received posterior tibial nerve (PTN) blocks to facilitate debridement and wound care.

Originating from the sciatic nerve, the PTN provides both motor and sensory input to the plantar aspect of the foot.^10^ Previous studies have demonstrated the accessibility and effectiveness of PTN blocks for calcaneal fractures and foreign body removal in pediatric patients.^3^–^6^ Nerve blocks at the stellate ganglion, lumbar epidural space, and at the distal nerve of the wrist have been shown to provide substantial pain relief prior to frostbite treatment.^1^,^2^,^11^ To our knowledge there is no documented case of the PTN block used for frostbite management, despite its ease of application and historically high success rates for pain control.^7^,^8^

## CASE SERIES

**Image 1:**
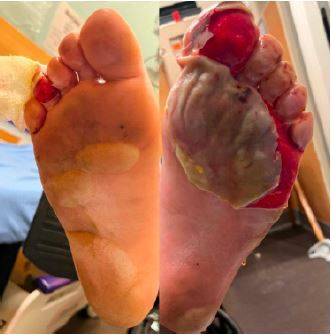
Bilateral partial-thickness frostbite on patient 1 with a broken serous blister on the left.

**Image 2:**
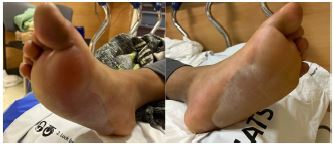
Bilateral partial-thickness frostbite present on patient 2. Large serous blisters are present.

All three of our patients presented with bilateral plantar surface frostbite injuries that occurred during the winter months. Patient 1 was a 17-year-old male with 4% total body surface area (TBSA) partial-thickness burns that occurred approximately 24 hours prior (Image 1). Patient 2 was an 18-year-old female with 5% TBSA partial-thickness burns from a more recent exposure (Image 2). Patient 3 was a homeless male with 4% TBSA partial-thickness burns who frequently walked outside barefoot. The patients reported their pain from 7/10 to 10/10 prior to pain medication, with only modest improvement after receiving oral analgesia. The specific oral analgesia given and subsequent pain scores are delineated in the Table.

**Image 3:**
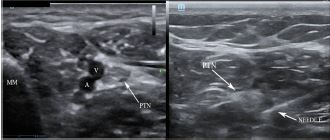
Posterior tibial nerve (PTN) block. Left demonstrates anatomical landmarks of the medial malleolus (MM), vein (V) and artery (A). Right, the needle with anesthetic spread around the PTN coming from a posterior approach.

Ultrasound-guided PTN blocks were performed using the same technique for each patient (Image 3), blocking the left PTN for patient 1 and bilateral PTNs for patients 2 and 3. Using a linear transducer, the PTN was identified adjacent to the medial malleolus, keeping the posterior tibial artery and vein in view. The needle was advanced using an in-plane approach and normal saline was introduced to confirm location at the nerve and away from surrounding structures. Following conformation, five milliliters of local anesthetic was injected and observed to surround the nerve. After 10 minutes the patients were re-evaluated, and each patient reported significant improvement in their pain, all scoring 0/10 on the pain scale. The table highlights the type of local anesthetic and post-block pain scores for each patient. Successful blister debridement was performed in the ED for patients 1 and 3. Patient 2 had her burn care performed on the burn surgery service floor immediately following PTN block in the ED. There were no reports of local or systemic toxicity from the anesthetic.

**CPC-EM Capsule**

What do we already know about this clinical entity?

*Frostbite injuries are debilitating, painful and require rapid identification and appropriate pain control and wound care to optimize outcomes*.

What makes this presentation of disease reportable?

*Data is limited on the use of the posterior tibial nerve (PTN) block to effectively provide pain control for frostbite injuries of the plantar surfaces*.

What is the major learning point?

*The PTN block can be used to provide pain control for patients with plantar surface frostbite in the emergency department*.

How might this improve emergency medicine practice?

*Emergency physicians can utilize the PTN block for plantar surface frostbites to improve pain control and wound management*.

Patient 1 was discharged home after debridement in the ED, while patients 2 and 3 were admitted for further management. None of the patients underwent repeat PTN blocks for wound care while outpatient or inpatient. Patient 1 was seen in the burn clinic three days after his ED visit. In that visit, the tissue of his left foot was pink and moist, and the team planned to debride a serous blister on his right foot. He was given oral (PO) oxycodone but was unable to tolerate the procedure due to pain. Patient 2 had multiple repeat debridements on the burn surgery service where IV opiate medications were utilized for pain management. She described these debriedments as “unbearable” and “so painful.” On hospital day #5, the patient required procedural sedation with propofol for proper dressing changes and additional debridement. Once she was able to tolerate dressing changes without intravenous pain medications, she was deemed safe for discharge. Patient 3 required a 10-day hospital admission for co-management of frostbite pain and alcohol withdrawal.

## DISCUSSION

This case series demonstrates the severity of frostbite pain and the challenge it creates to receiving appropriate wound care. Ultrasound-guided PTN blocks bypass this challenge and achieve effective analgesia in the ED, allowing for optimal wound debridement as shown in these patients’ experiences.

There are multiple strengths and limitations in our approach. The most clinically relevant benefit of the PTN block is the analgesia it provides. Pain is a significant barrier to treating frostbite injuries, originating from the burn itself as well as from rewarming and debridement.^2^,^9^ All patients reported pain scores of 7/10 to 10/10 with PO medications only. Following the PTN block, patients reported drastic improvement in their pain levels and tolerated debridement without additional medications. This outcome aligns with prior studies that have shown pain control success rates of 95–100% when using the PTN block for foot surgeries.^7^,^8^ Later attempts at repeat debridement were either unsuccessful or performed under procedural sedation for patient one and patient two, highlighting the superiority of the PTN block analgesia.

The addition of ultrasound guidance further improved the PTN block. Peripheral nerve blocks performed with ultrasound provide better pain control, require fewer additional pain medications, and have fewer complications as compared to landmark guidance.^4^,^12^,^13^ A study by Kakhi et al revealed shorter time to onset and longer duration of analgesia with ultrasound guidance.^13^ Shah et al demonstrated the superior accuracy of ultrasound for targeting the posterior tibial nerve and avoiding surrounding structures.^14^ This accuracy translates into increased block success and fewer incidents of intravascular injection and systemic toxicity.^4^,^13^,^14^ Each of our patients reported significant pain relief that was achieved in less than 10 minutes, and no patient experienced adverse effects.

Our experience using the PTN block for these patients demonstrates the accessibility and relevance of this block for future patient encounters. The PTN block is a well described and thoroughly examined nerve block that is accessible to clinicians of different training levels and experience.^7^,^8^,^15^ In the series we report, it was performed by first- and third-year emergency medicine residents under the guidance of an ultrasound-trained faculty member. Emergency departments in colder climates frequently see frostbite as a chief complaint, and this case series can help guide the use of PTN blocks for pain control in such patients.

Despite the accessibility of the PTN block, clinician comfort with nerve blocks and the availability of ultrasound-trained faculty could limit its use. The ultrasound-guided block is also more time intensive when compared to PO or IV pain medications.^4^ Lastly, our case series only focuses on the experiences of three patients with second-degree frostbite of bilateral plantar surfaces. Patients with other levels and locations of frostbite injury may have different outcomes.

## CONCLUSION

This case series demonstrates that the ultrasound-guided PTN block provides superior pain relief, has low risk of systemic toxicity, allows for necessary wound care, and is accessible to clinicians of varying training levels. Ultrasound-guided PTN blocks have the potential to play a major role within a multi-modal pain control strategy for frostbite management. This role is unequivocally applicable within the emergency department. The ease and accessibility of the block also lends its use to other clinical contexts, including outpatient wound clinics and inpatient burn units. These potential applications of the PTN block warrant further research on its use for frostbite management in different clinical scenarios.

## Figures and Tables

**Table:** Patient pain scores after oral analgesia and after posterior tibial nerve block.

Patient	% TBSA burn	PO medications	Pain score after PO medications	Local anesthetic used for PTN block	Pain score after PTN block
1	4%	Acetaminophen, ibuprofen, oxycodone, olanzapine	6/10 – 10/10	5 mL 0.25% bupivacaine	0/10
2	5%	Acetaminophen, ibuprofen, oxycodone	7/10	5 mL 0.5% ropivacaine	0/10
3	4%	Ibuprofen	10/10	5 mL 0.25% bupivacaine	0/10

*TBSA*, total body surface area; *PO*, oral medication; *PTN*, posterior tibial nerve; *mL*, milliliter.
